# Decoding of Ankle Joint Movements in Stroke Patients Using Surface Electromyography

**DOI:** 10.3390/s21051575

**Published:** 2021-02-24

**Authors:** Afaq Noor, Asim Waris, Syed Omer Gilani, Amer Sohail Kashif, Mads Jochumsen, Javaid Iqbal, Imran Khan Niazi

**Affiliations:** 1 Department of Biomedical Engineering & Sciences, School of Mechanical & Manufacturing Engineering, National University of Sciences and Technology (NUST), Islamabad 44000, Pakistan; afaqahmed.pg@smme.edu.pk (A.N.); omer@smme.nust.edu.pk (S.O.G.); amer.kashif@smme.nust.edu.pk (A.S.K.); j.iqbal@ceme.nust.edu.pk (J.I.); 2 Department of Health Science and Technology, Aalborg University, 9220 Aalborg Øst, Denmark; mj@hst.aau.dk; 3 Center for Chiropractic Research, New Zealand College of Chiropractic, Auckland 1060, New Zealand; 4 Health and Rehabilitation Research Institute, AUT University, Auckland 1010, New Zealand

**Keywords:** stroke rehabilitation, surface electromyography (sEMG), pattern recognition (PR), ankle joint movements, home-based physical therapy, lower limb functional recovery

## Abstract

Stroke is a cerebrovascular disease (CVD), which results in hemiplegia, paralysis, or death. Conventionally, a stroke patient requires prolonged sessions with physical therapists for the recovery of motor function. Various home-based rehabilitative devices are also available for upper limbs and require minimal or no assistance from a physiotherapist. However, there is no clinically proven device available for functional recovery of a lower limb. In this study, we explored the potential use of surface electromyography (sEMG) as a controlling mechanism for the development of a home-based lower limb rehabilitative device for stroke patients. In this experiment, three channels of sEMG were used to record data from 11 stroke patients while performing ankle joint movements. The movements were then decoded from the sEMG data and their correlation with the level of motor impairment was investigated. The impairment level was quantified using the Fugl-Meyer Assessment (FMA) scale. During the analysis, Hudgins time-domain features were extracted and classified using linear discriminant analysis (LDA) and artificial neural network (ANN). On average, 63.86% ± 4.3% and 67.1% ± 7.9% of the movements were accurately classified in an offline analysis by LDA and ANN, respectively. We found that in both classifiers, some motions outperformed others (*p* < 0.001 for LDA and *p* = 0.014 for ANN). The Spearman correlation (ρ) was calculated between the FMA scores and classification accuracies. The results indicate that there is a moderately positive correlation (ρ = 0.75 for LDA and ρ = 0.55 for ANN) between the two of them. The findings of this study suggest that a home-based EMG system can be developed to provide customized therapy for the improvement of functional lower limb motion in stroke patients.

## 1. Introduction

Stroke remains one of the leading causes of social isolation, disability, and death [1]. In children, the incidence of stroke is rare [2] and it has been estimated that in both men and women, the risk of stroke increases with age [3] while women have more stroke events than men [4].

As the average age of population is increasing across the world due to multiple reasons such as advances in medical technology, health care system, and provision of cheap and readily available medicines, it is expected that the number of stroke patients will rise [5,6]. Consequently, more patients will need physical rehabilitation in the future and governments will require induction of an increased number of healthcare professionals than usual to provide physical rehabilitation to these individuals. It is also more likely that the economic burden of stroke will increase and pose challenges to those health systems with limited resources [7].

A stroke survivor faces long-term effects after the acute phase of stroke. These effects include the development of impairment, limitations of activities (disability), and reduced participation (handicap) [8]. Although stroke results in a variety of physical and cognitive abnormalities, the most widely recognized is motor impairment, which affects 80% of stroke patients [9]. Commonly, stroke results in loss of movement control of one side of the body, impacting locomotion.

In neurological disorders including stroke, the restoration of physical function heavily depends on the onset, the type of injury, and the paradigm being followed for motor function recovery [10]. For the rehabilitation of upper limb motor function in stroke patients, constraint-induced movement therapy (CIMT), robotics, brain–computer interfaces (BCIs), electromyographic biofeedback, and mental practice (MP) combined with motor imagery have shown improvements in motor function [11,12,13,14]. Additionally, repetitive task training, high intensity physiotherapy/physical therapy (PT) and PT in combination with MP [15] have resulted in improved functional outcomes for lower limb mobility [16].

Assessing the outcomes of PT over time is very important in evaluating the functional performance of patients as well as the administered intervention. There are many scales used to access motor performance after stroke, but the most commonly used scale for assessing motor impairments in clinical practice is the Fugl-Meyer Assessment (FMA) scale [17].

The use of the upper limb is more frequent in performing activities of daily life (ADLs) and the upper limb has been targeted vastly in the areas of physical rehabilitation. Currently, there are various commercially available and widely used rehabilitative systems for upper limb rehabilitation after stroke, such as exoskeletons [18], rehabilitation robots [19], gaming devices [20], and virtual reality (VR) based systems [21]. Many of these rehabilitative devices are electromyography (EMG)-based. However, there is no device commercially available and clinically proven for stroke patients with lower limb motor impairments. Some of the main challenges hindering the commercial availability of the many proposed lower limb rehabilitative devices include design limitations and do not account for the physical requirements of stroke patients [22]. Additionally, more research focus on upper limbs is also an important factor that has resulted in researchers’ comparatively less technical inclination toward the development of lower limb rehabilitative devices.

The first step toward the development of an EMG-controlled and home-based lower limb motor rehabilitation device is to investigate the movements of the lower limb in stroke patients. In the normal functionality of the lower limbs, movements that occur at the ankle joint complex have major significance in gait and balance. The available literature on the decoding of ankle joint motions from the movement intention of a user using surface electromyography (sEMG) in healthy subjects as well as in stroke patients is limited. However, Al-Quraishi et al. [23] successfully decoded ankle joint movements in healthy subjects while investigating the impact of different feature extraction and dimensionality reduction techniques on classification accuracies using autoregressive (AR) features and the following classifiers: K-nearest neighbor (k-NN), multilayer perceptron (MLP), and linear discriminant analysis (LDA). Their findings suggested that k-NN along with fuzzy neighborhood preserving analysis with QR (FNPA-QR) decomposition, as a dimensionality reduction technique, provides superior results with an average accuracy of 96.20% ± 4.1%. In another study exploring the biomechanical strategies used by healthy individuals during walking over uneven terrain, Gregory et al. [24] utilized time domain (TD) features (second-order AR coefficients, integrated EMG (IEMG), variance (VAR), waveform length (WL), moving average, and root mean square (RMS)) to predict user intent of performing ankle joint motions using LDA and the classification tree (CART) from sEMG signals. They reported the highest classification accuracy of 77.2% using LDA. Furthermore, Waris et al. [25] evaluated six different classifiers (LDA, ANN, K-NN, SVM, TREE, and naïve Bayes) in a multiday evaluation to identify the most suitable algorithm for sEMG classification of hand motions. In their study, ANN performed better of all classifiers.

The purpose of this study was to investigate the potential use of sEMG for a home-based ankle joint rehabilitative device using PR approaches and to evaluate the performance of two classifiers (ANN and LDA). In this study, the intent of performing different ankle joint movements was decoded from the recorded sEMG of stroke patients and the relationship between motor impairment and functional movements was explored. Previously, it has been reported that the classification accuracy for upper limbs is affected by impairment level in stroke patients [26,27]. Additionally, patients were observed by the data collection team throughout the experimental protocol and notes were taken. A total of four movements take place at the ankle joint complex: dorsiflexion and plantar flexion in the sagittal plane, and eversion and inversion in the frontal plane [27].

## 2. Materials and Methods

### 2.1. Participants

Fourteen stroke patients participated in this study from Railway General Hospital in Rawalpindi, Pakistan. All the participants were male. Only those patients who had a Mini-Mental State Examination (MMSE) score of greater than 24 were included in this study [28], which means they did not suffer from any severe cognitive disorder and were able to understand the given instructions. Based on the mentioned criteria, three patients were dropped (see the patient demographics in Table 1). An informed consent was provided by all patients prior to their participation in this study. This study was carried out according to the rules of the Declaration of Helsinki of 1975 and the experimental protocol for this study was approved by the local ethical committee (Riphah /RCRS /REC /00651). The Fugl-Meyer Assessment was carried out by a registered Physical Therapist to provide the scores for motor impairment. The motor section of the Fugl-Meyer Assessment scale consists of 100 points in total, which are used for the assessment of motor function in the upper extremity (66 points) and lower extremity (34 points) [29].

### 2.2. Recordings: Surface EMG

Six surface EMG electrodes (Ambu Neuroline 720 surface electrodes, REF 72000-S/25, Baltropbakken 13, DK-2750, Ballerup, Denmark) were placed on the paretic leg on Tibialis anterior, gastrocnemius, and peroneus longus [23]. Two electrodes were placed in bipolar configuration 2 cm apart on the belly of each muscle to acquire signal from a given muscle. A moist wristband was used as a reference for signals. The sampling frequency was 2048 Hz, and the signals were amplified with a gain of 10,000 (EMG-USB2+, OT-Bioelettronica Metropolitan City of Turin, Italy).

### 2.3. Experimental Setup

**Figure 1:**
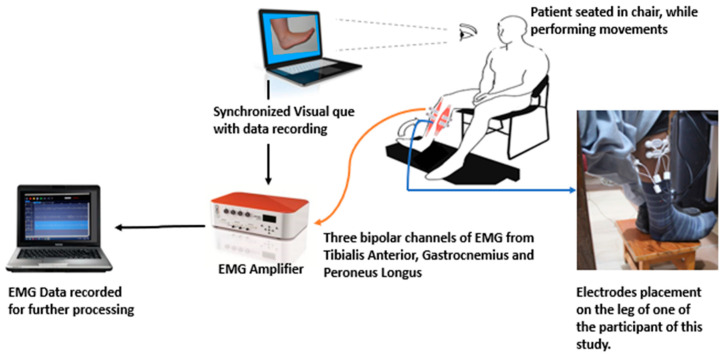
An illustration of the electromyography (EMG) data recording setup while the patient was performing movements.

Initially, the sEMG electrodes were placed on the affected leg and the quality of the signals were examined. The experimental protocol was explained in detail to each participant and a visual cue (photo of each motion class) was used to guide the subjects during the experiment (Figure 1). Furthermore, each subject was first asked to perform ankle joint movements with both limbs to familiarize them with the experimental protocol. Each subject performed one recording session while seated in a comfortable chair. A digital trigger was transmitted to the amplifier both at the beginning and at the end of the recording session so that the EMG recordings and the visual cue were synchronized. The following motion classes were included: plantar flexion, dorsiflexion, eversion, inversion, and rest. The time duration of performing a single instance of motion was six seconds (each patient was told to sustain the muscle contraction for six seconds) and every movement was repeated ten times. A rest time of six seconds was included between each repetition. The order of the motion classes was randomized and after completion of one motion class, the next motion class began.

## 3. Data Analysis

### 3.1. Pre-Processing and Feature Extraction

**Figure 2:**
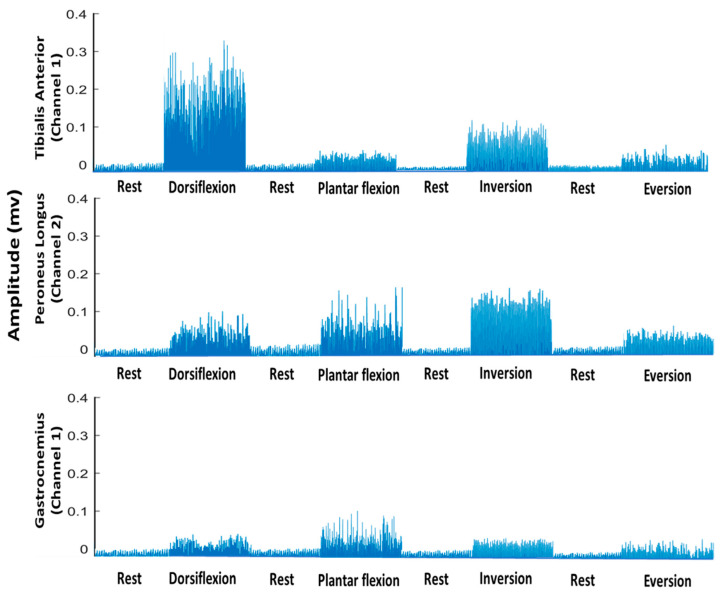
Rectified and bandpass-filtered EMG signal (only for understanding and visualization) of all motion classes and all channels separately for subject 3.

A bandpass filter with cut-off frequencies of 20 and 500 Hz and a Notch filter from 49–51 Hz were applied to the EMG signals using a second-order Butterworth filter with no phase shift to remove noise. Every six second repetition of each motion class was extracted and the first and last seconds were excluded from the analysis. As a result, 4 s epochs for every repetition of each motion class were obtained. After pre-processing, the following five (TD) features were extracted: ZC, WL, MAV, SSC [29] and Wilsons amplitude (WAMP) [30,31]. A 280 ms overlapping data window with a step size of 20% was used for feature extraction [32]. Figure 2 shows an example of a rectified and filtered EMG signal for every motion class and the associated channels (muscles) used for recording the EMG.

### 3.2. Classification

LDA and ANN (1 hidden layer and 15 neurons) were used to classify the different lower limb motions. The classification problem consisted of 5 classes. In this analysis, a four-fold cross-validation was used and the data were randomly divided into four folds for both the classifiers. Furthermore, one fold was used for testing while the remaining three folds were used to train the classifier. The overall average across the four folds is reported. Within the session, calibration was used to perform the classification, i.e., the training and testing were performed on the same session’s data. All the extracted features were included in the analysis. MATLAB R2020a (MathWorks^®^, Natick, MA, USA) was used to perform data processing and classification. All the analyses were performed on a laptop with a 64-bit operating system, a core i5 processor, and 8 GB RAM.

### 3.3. Statistics

One-way repeated measures of analysis of variance (ANOVA) were used for separate inferential statistical analysis of the classification results of ANN and LDA to investigate the performance variance in the different motion classes. A probability value (*p*-Value of < 0.05) was used to infer the significance of all statistical analysis. Significant results were followed by a post-hoc test with Bonferroni correction for multiple comparisons. Lastly, the Spearman correlation coefficient (ρ) and coefficient of determination (R^2^) were calculated between the lower limb Fugl-Meyer score and the average class accuracies of ANN and LDA for individual motions, as well as for all motions combined. All statistical analyses were performed using IBM^®^ SPSS^®^, Chicago, IL, USA).

## 4. Results

**Figure 3:**
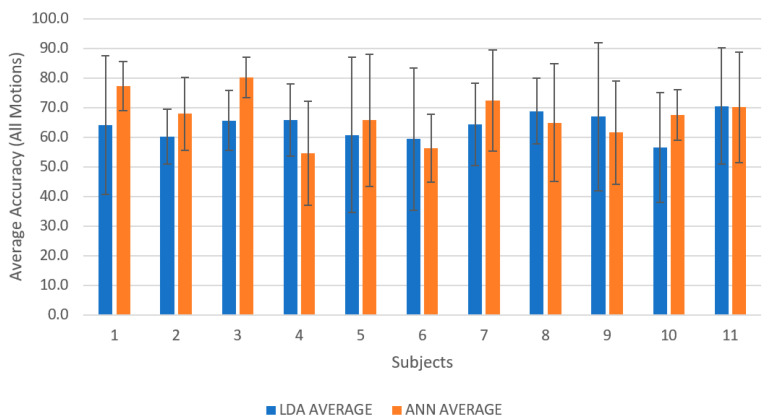
A column graph representing the average classification accuracies for all subjects using linear discriminant analysis (LDA) and artificial neural network (ANN) reported in the form of mean ± standard deviation.

**Figure 4:**
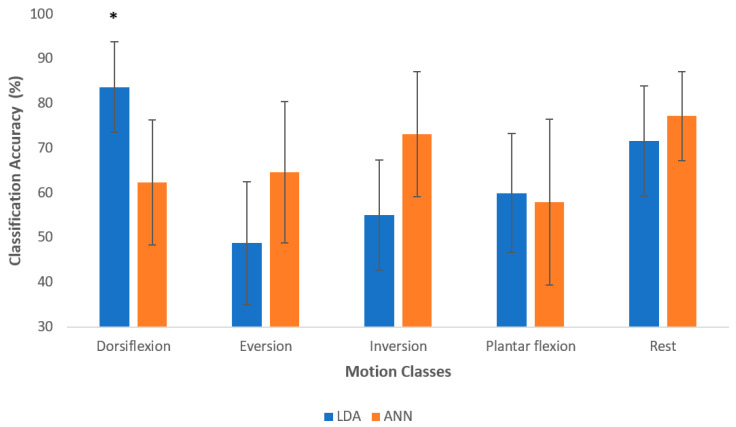
The average classification accuracy for all motion classes across all participants. The results are reported as mean ± standard deviation and asterisk denotes significant differences between motion class for each classifier. The asterisk denotes statistically significant difference.

**Figure 5:**
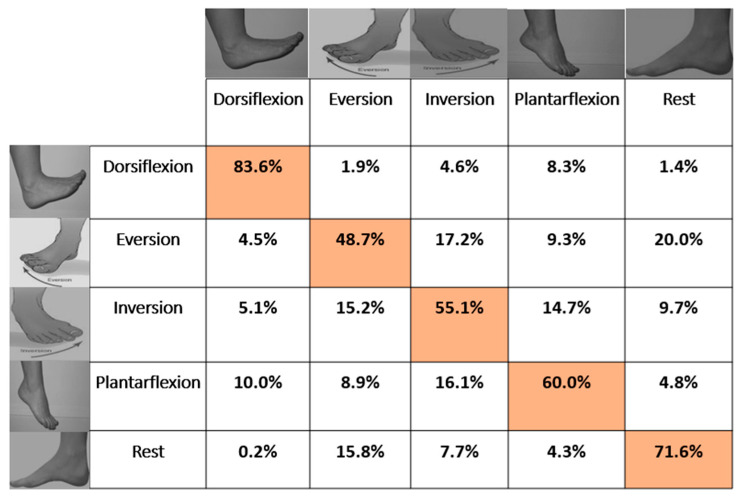
Confusion matrix based on the average classification accuracies of all subjects for LDA with overall average accuracy of 63.86% ± 4.3%. The Highlighted boxes represents the correct percentage of predictions made by the classifier.

**Figure 6:**
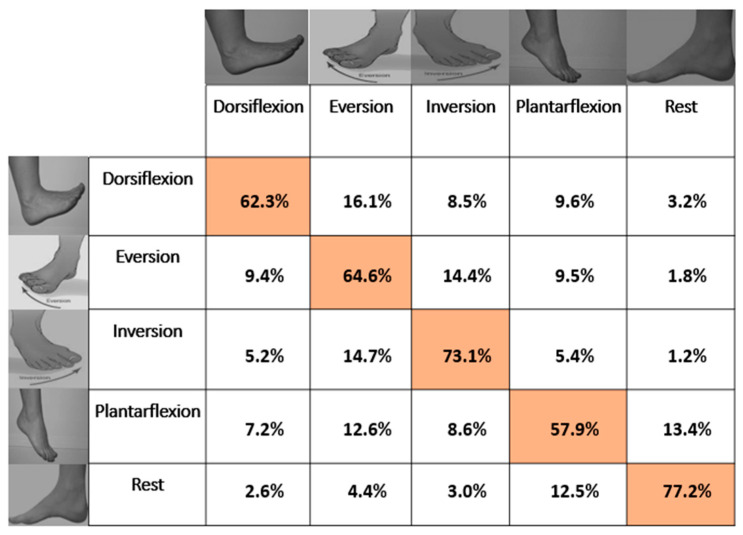
Confusion matrix based on the average classification accuracies of all subjects for ANN with overall average accuracy of 67.1% ± 7.9%. The Highlighted boxes represents the correct percentage of predictions made by the classifier.

**Figure 7:**
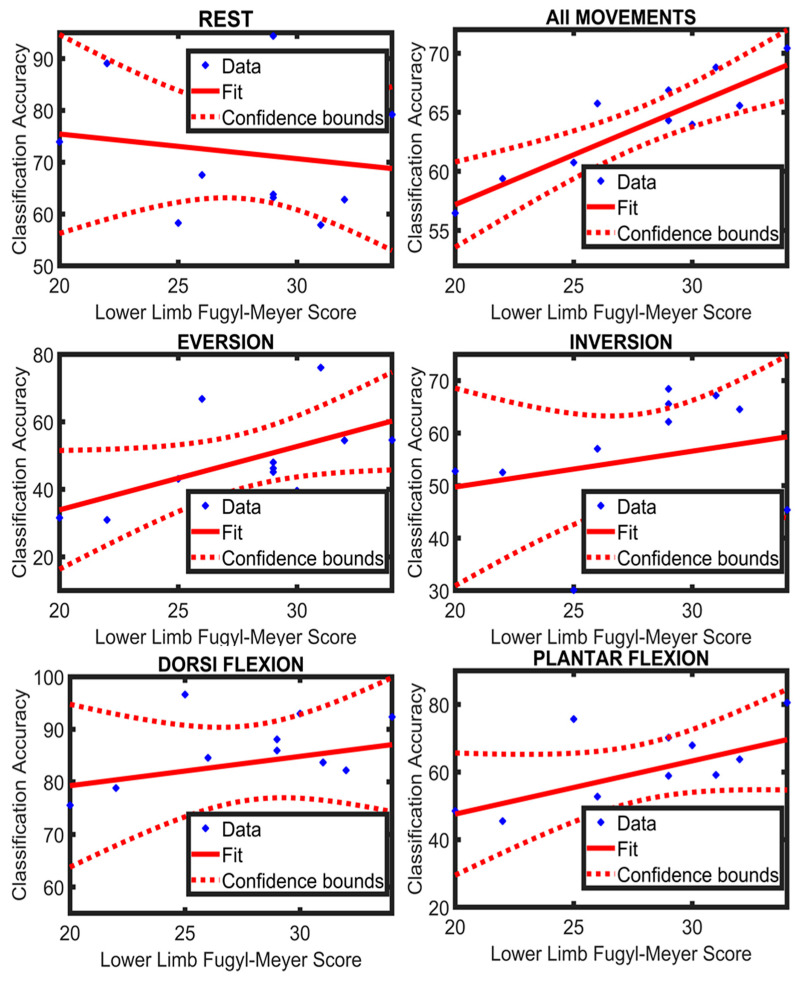
A regression line fitted to the participants’ data of Limb Fugl-Meyer score and their classification accuracies (LDA) of individual movement and all movements combined.

**Figure 8:**
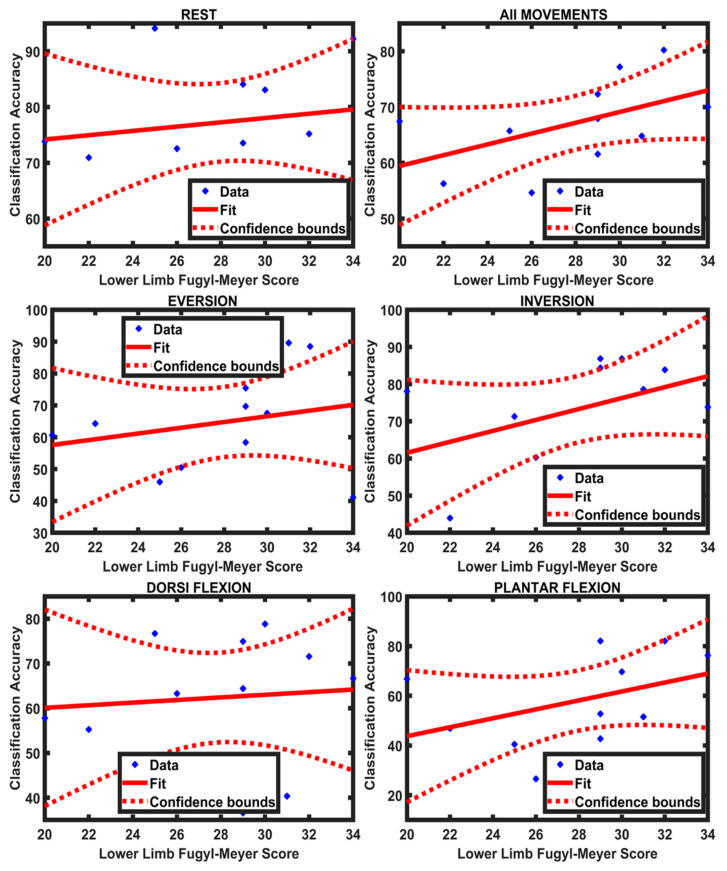
A regression line fitted to the participants’ data of Limb Fugl-Meyer score and their classification accuracies (ANN) of individual movements and all movements combined.

The results of this study related to motions classification and the association between level of motor impairment and its effect on performing ankle joint movements are summarized in Table 2 and in Figure 2, Figure 3, Figure 4, Figure 5, Figure 6, Figure 7 and Figure 8. As studies have reported differences in EMG signals on the basis of sex, age, and anthropometric variables; therefore, the results of this study are not representative of the whole population [33].

### 4.1. Classification Results

On average across all participants, 63.85% ± 4.2% and 67.09% ± 7.8% of the movements were accurately classified in the offline analysis using LDA and ANN, respectively. One-way repeated measures of analysis of variance (ANOVA) revealed a significant difference between motion classes for both LDA ((*F* (4, 40) = 12.48; *p* < 0.001; partial η^2^ = 0.55) and ANN ((*F* (4, 40) = 3.57; *p* < 0.001; partial η^2^ = 0.26). For LDA, the post-hoc test with Bonferroni correction for multiple comparisons revealed that dorsiflexion was significantly different from eversion, inversion, and plantar flexion. Additionally, using LDA, dorsiflexion was the easiest to discriminate (83.6%). The accuracies of the other motion classes were in the range between 48% and 72%. The accuracies of dorsiflexion and the rest class were higher than all other motion classes, while the accuracies of plantar flexion and inversion were higher than that of eversion. The confusion matrix in Figure 5 shows that rest and eversion were mostly confused by LDA. In the classification using ANN, rest was classified most accurately with an accuracy of 77.1%, and plantar flexion had the lowest accuracy of 57.9%. For ANN, the post-hoc test with Bonferroni correction for multiple comparisons revealed that dorsiflexion was significantly different from rest.

On average, ANN outperformed LDA (see Figure 3 showing subject-wise results), and similarly in motion-wise results, the mean classification accuracy of ANN was better than LDA; however, LDA identified dorsiflexion and plantar flexion better than ANN. In addition, there was a significant difference between LDA and ANN when identifying dorsiflexion (*F* (9, 90) = 7.07; *p* < 0.001; partial η^2^ = 0.414). Figure 4 reports the average classification accuracy for all the motion classes across all participants in the form of mean ± standard deviation. The confusion matrices (mean across the movements and across participants) are also shown in Figure 5 and Figure 6 for LDA and ANN, respectively.

### 4.2. Relationship between Functional Score and Classification Accuracy

The Spearman correlation was calculated between the lower limb FMA score (section E-F of FMA scale) and the class accuracy across all movements as well as each movement individually for ANN and LDA separately. Furthermore, the coefficient of determination (R^2^) was also calculated between the lower limb FMA score and the classification accuracy of all movements and each movement separately for both the classifiers. There was a positive, moderate association between the functional scores and the classification accuracies of the motion classes; the results for the correlation analysis and coefficient of determination are presented in Table 2. Additionally, a regression line was fitted to the values of the lower limb FMA score and the average classification accuracy of LDA and ANN for all movements and each movement individually (Figure 7 and Figure 8). It can be observed from Table 2 that the value of R^2^ for all movements is greater in LDA (R^2^ = 0.71) compared to ANN (R^2^ = 0.27). This suggests that LDA was better at identifying a possible trend between the classification accuracies and the lower limb FMA scores.

### 4.3. Patients’ Feedback

For the participating stroke patients, data recording via sEMG was a new experience. At the end of every recording session, each patient was asked to share their experience with the computer-guided training, and 9 among the 11 participants responded positively. Table 3 provides the questions that were posed to each subject at the end of the recording session along with their answers. Their positive response was foreseeable because of the interactive nature of the experimental protocol.

## 5. Discussion

The aim of this study was to investigate the potential use of sEMG as a controlling mechanism for a home-based lower limb rehabilitative device for use in post-stroke PT. In this study, we were able to decode five different motion classes: dorsiflexion, eversion, inversion, plantar flexion, and rest (also included as a motion class) in stroke patients with an average accuracy of 63.86% ± 4.3% and 67.1% ± 7.9% using LDA and ANN, respectively. We found that in both classifiers, some motions outperformed others (*p* < 0.001 for LDA and *p* = 0.014 for ANN). The results revealed that ANN performed better than LDA, which is in agreement with a recent study aimed at characterizing distinct motions in healthy subjects and amputees using sEMG and intramuscular electromyography (IEMG) signals [34].

From earlier sources, it was known that ankle joint movements can be decoded from the recorded sEMG data of healthy subjects [23]. Furthermore, it was reported that the intent of performing lower limb movement (as decoded from sEMG signals) can be used to control a rehabilitative device [35]. We were able to successfully decode ankle joint movements from recorded sEMG data of stroke patients. The findings of the current study are in accordance with previous studies indicating that EMG activity of attempted movements can be decoded from stroke patients with motor impairments [26,36,37,38,39,40]. However, the number of sEMG channels used in the current setup was less than in other studies [40,41]. Hudgins (TD) features [30,31,42,43,44] were used in this study, but adding other features derived from autoregressive coefficients or wavelets and increasing the number of sEMG channels will probably improve the classification accuracy

In the current analysis, we found a positive and moderate association between the extent of motor impairment (lower limb FMA score) and average classification accuracies for both the classifiers, indicating that as the classification accuracy increases (in case of both LDA and ANN) moderately, there is small increase in the lower limb FMA score. This is in accordance with a previous study, which reported that the classification accuracies decrease as a function of the severity of stroke [39]. It is more likely that a perfect association was not found because of the small sample size (limited number of stroke patients) and the possibility that the lower limb FMA score is not a very sensitive measure for quantifying the level of impairment in these movements specifically, since the FMA score also includes reflexes, and muscle synergies, knee movements, etc.

During our investigation, we asked patients to perform various lower limb movements in order to familiarize themselves with the experimental protocol. We observed that patients felt relaxed when they were asked to perform the same movement using both limbs simultaneously. This can be attributed to the interaction between the paralyzed and the normal cerebral cortex, which results in additional stimulation [45]. It is also known that exercising both the normal and the affected side in stroke patients is more effective in upper limb function recovery compared to exercising the affected side alone [46]. New studies involving lower limbs can improve current knowledge on this factor. It was also observed that patients seemingly performed movements well when the recording session was closer to ending, which means that some level of dexterity can be achieved in performing these motions within only one session of training, but this needs to be further explored in a specially designed experiment.

For lower limb rehabilitation after stroke, manual PT is mostly used in clinical practice [47,48]. However, neuro-rehabilitation with the help of robotic devices presents a bright future [49,50,51]. These devices facilitate personalized treatment, offer reliable assessments, and ease PT from rigorous manual therapy [52,53,54]. Based on mechanical design and actuation, there are two categories of ankle rehabilitation device: end-effector-based robots and robotic exoskeletons/orthoses [55]. Furthermore, many lower limb rehabilitation devices have been developed in the past two decades, but most of these mechatronic systems use a combination of force sensors and feedback signals from position encoders. Besides, sEMG has been successfully used as a controlling mechanism for many upper limb rehabilitation devices but rarely investigated for lower limb rehabilitative devices [56]. Despite the successful use of sEMG for upper limb rehabilitative devices, its potential has not been studied extensively for lower limb PT devices.

The possibility of using sEMG as a controlling mechanism for a home-based rehabilitative device was demonstrated in this pilot study. In the current investigation, the trial arrangement was moderately cumbersome. However, the use of compact sEMG technologies such as the Myo-armband can make the use of sEMG much easier for patients. Furthermore, it was shown that comparable accuracies can be achieved using such setups [57]. New studies should be carried out involving a large sample size and smaller setups to validate these findings in the future.

Presently, because stroke patients are at great risk to coronavirus disease (COVID-19) [58], their rehabilitation regimen was severely affected in the months following the pandemic. The possibility of unavoidable circumstances like COVID-19 also advocates for shifting the focus of stroke-related research toward home-based PT devices so that in the future, stroke patients can continue their PT even if physical therapists are not available or the therapy is not feasible. Many systems have been introduced for the rehabilitation of the ankle joint, but the majority of these systems cannot be used in lower limb rehabilitation after stroke [22]. A dire need exists to address the challenges encountered in the development of EMG-based lower limb rehabilitation devices and the promotion of home-based PT for stroke patients.

## 6. Conclusions

It is possible to decode various motion classes of the ankle joint in stroke patients using a PR-based technique (LDA and ANN) requiring a smaller experimental setup and offering a high level of classification performance, which is a key factor for the efficacy of home-based rehabilitative devices. However, the availability of the number of stroke patients was limited and we propose that more studies are needed to validate these findings with a bigger sample size of stroke patients. Furthermore, from the current study, we concluded that research involving experts from different areas and an interdisciplinary approach is more fruitful and viable.

## Figures and Tables

**Table 1:** Demographics of the patients who participated in this study.

Patient No.	Age	Sex	Months Since Injury	Affected Side	Injury	Fugl-Meyer Score of Lower Limb (E-F)
1	59	Male	15	Right	Ischemic	30
2	61	Male	7	Right	Ischemic	29
3	50	Male	35	Right	Ischemic	32
4	56	Male	8	Left	Ischemic	26
5	49	Male	20	Right	Ischemic	25
6	58	Male	12	Right	Ischemic	22
7	62	Male	34	Left	Ischemic	29
8	48	Male	8	Left	Hemorrhagic	31
9	57	Male	60	Right	Hemorrhagic	29
10	60	Male	20	Left	Ischemic	20
11	40	Male	40	Left	Hemorrhagic	34

**Table 2:** Values of the Spearman correlation (ρ) and coefficient of determination (R^2^) analysis between classification accuracies and movements (individual movement and average of all movements).

Motions	LDA			ANN		
	ρ	(*p*-Value)	R^2^	ρ	(*p*-Value)	R^2^
All Movements	0.75	<0.001	0.71	0.55	0.07	0.27
Dorsiflexion	0.26	0.42	0.05	0.21	0.53	0.00787
Eversion	0.64	0.03	0.34	0.28	0.39	0.05
Inversion	0.22	0.49	0.05	0.43	0.18	0.2
Plantar flexion	0.51	0.1	0.25	0.54	0.08	0.17
Rest	−0.15	0.64	0.03	0.12	0.7	0.02

**Table 3:** Set of questions that were posed to each subject at the end of recording session and each subject’s response.

Q.	Questions (Total Participants 11)	Yes	No
1	Have you ever participated in a scientific study like this one?	0	11
2	Was it convenient for you to follow the series of images and perform the exercise?	11	0
3	Do you want to participate in another session?	10	1
4	Do you feel in control while doing the exercise on your own without the help of a Physical therapist?	10	1
5	Did you feel fatigued?	1	10
6	Are you in favor of a rehabilitative device that will provide physical therapy in your own environment?	10	1
7	Did you feel pain at any time during the experimental protocol?	0	11
8	Did you feel relaxed during the experiment?	10	1

## Data Availability

The data for this study has been archived at the Department of Biomedical Engineering, School of Mechanical and Manufacturing Engineering (SMME), National University of Sciences and Technology (NUST), Islamabad, Pakistan. The data is private property of NUST. The data is available on request as per the University Policy.
